# A deeper view into the significance of simple sequence repeats in pre-miRNAs provides clues for its possible roles in determining the function of microRNAs

**DOI:** 10.1186/s12863-018-0615-x

**Published:** 2018-05-09

**Authors:** Nisha Joy, Y. P. Maimoonath Beevi, E. V. Soniya

**Affiliations:** Plant Disease Biology and Biotechnology, Rajiv Gandhi Center for Biotechnology, Poojappura, Thiruvananthapuram, Kerala 695014 India

**Keywords:** Non-coding RNAs, Tandem repeats, Pre-miRNAs, Alternative splicing, *Arabidopsis thaliana*

## Abstract

**Background:**

The central tenet of ‘genome content’ has been that the ‘non-coding’ parts are highly enriched with ‘microsatellites’ or ‘Simple Sequence Repeats’ (SSRs). We presume that the presence and change in number of repeat unit (n) of SSRs in different genomic locations may or may not become beneficial, depending on the position of SSRs in a gene. Very few studies have looked into the existence of SSRs in the hair-pin precursors of miRNAs (pre-miRNAs). The interplay between SSRs and miRNAs is not yet clearly understood.

**Results:**

Considering the potential significance of SSRs in pre-miRNAs, we analysed the miRNA hair-pin precursors of 171 organisms, which revealed a noticeable (29.8%) existence of SSRs in their pre-miRNAs. The maintenance of SSRs in pre-miRNAs even in the complex, highly evolved phyla like Chordata and Magnoliophyta shed light upon its diverse functions. Putative effects of SSRs in either regulating the biogenesis or function of miRNAs were more underlined based on computational and experimental analysis. A preliminary computational analysis to explore the relevance of such SSRs maintained in pre-miRNA sequences led to the detection of splicing regulatory elements (SREs) either in or near to the SSRs. The absence of SSRs correspondingly decreased the detection of SREs.

**Conclusion:**

The present study is the first implication for the possible involvement of SSRs in shaping the SREs to undergo Alternative Splicing events to produce miRNA isoforms in accordance with different stress environments. This part of work well demonstrates the importance of studying such consistently maintained SSRs residing in pre-miRNAs and can enhance more and more research towards deciphering the exact function of SSRs in the near future.

**Electronic supplementary material:**

The online version of this article (10.1186/s12863-018-0615-x) contains supplementary material, which is available to authorized users.

## Background

The secret behind the difference in complexity of genome from small worms to highly evolved humans resides on the ‘non-coding’ part of the genome which was once considered as ‘dead ends’ or ‘genetic waste’. Reports point out that there is no proportional increase in the number of genes corresponding to the increase in complexity of the genome size, suggesting the evolution under positive selection pressure for the non-coding part of the genome. New high throughput sequencing technologies gave way to understand the importance of ‘non-coding transcripts’ and left behind the so far studied ‘coding transcripts’ that constitutes less percentage. The non-coding region includes two parts-the unique elements (promoters, enhancers, repressors, boundary elements, introns, conserved regions, pseudogenes, non-coding RNAs) and repetitive elements (transposable elements, tandem repeats) [1]. ‘Microsatellites’ or ‘Simple Sequence Repeats’ (SSRs) or ‘Simple Tandem Repeats’ (STR) are a major class of tandem repeats. They are tandem arrays of short (1–5 bp), repeated DNA sequences [2], that are commonly found in most genomes with a high mutation rate of 10^− 2^ to 10^− 6^ nucleotides per locus per generation [3] and hence utilized for fingerprinting studies. But once the discovery that change in tandem repeat unit of SSRs that fall in genes caused phenotypic changes, SSRs became more noticeable. The effect of SSRs were studied best across different plant species like rice [4, 5], common bean [6], barley [7], *Arabidopsis* [8] and also in humans [9].

But SSRs are poorly analysed in functional non-coding small regulatory RNAs like microRNAs (miRNAs). The importance of miRNAs (~ 20 nt) is that they play a major role in many biological processes and their biogenesis occurs from primary miRNA transcripts known as pri-miRNAs. The pri-miRNAs will adopt a stem-loop secondary structure known as the pre-miRNAs, from which a specific 21-nucleotide miRNA duplex is excised by a Dicer endonuclease [10]. Our previous experiments on transcriptome profiling revealed about the existence of SSRs in the non-coding transcripts of black pepper [11]. This true fact about the existence of SSRs in pre-miRNAs made us to ponder the possibility of SSRs in all the available pre-miRNAs across different taxa. To date, there is no lucid demonstration to prove the presence or pivotal functions of SSRs in hair-pin precursors of miRNAs, except for a few [11–13]. Hence our objective was to illustrate the exact incidence ratio of SSRs in all the available pre-miRNAs including plants, animals and viruses by performing a computational analysis in order to achieve a better understanding about the significance of such SSRs occurring in the pre-miRNAs. The preliminary observations revealed the significant incidence of SSRs and indicated the possible involvement of SSRs in Alternative Splicing (AS) events. AS also known as differential splicing is a regulated process that increases an organism’s transcriptome and proteome diversity [14]. One of the key regulators of AS are the cis acting Splicing Regulatory Elements (SREs) which are categorized into four classes like ESE (Exon Splicing Enhancer), ESS (Exon Splicing Silencer), ISE (Intron Splicing Enhancer) and ISS (Intron Splicing Silencer) depending on their location and its effect on splicing either as enhancers or silencers [15]. Here, the detection of SREs near SSRs in pre-miRNAs strongly suggests the possible involvement of SSRs in shaping episodes of AS.

## Results

### The distribution pattern of SSRs in hair-pin precursors of miRNAs

**Fig. 1:**
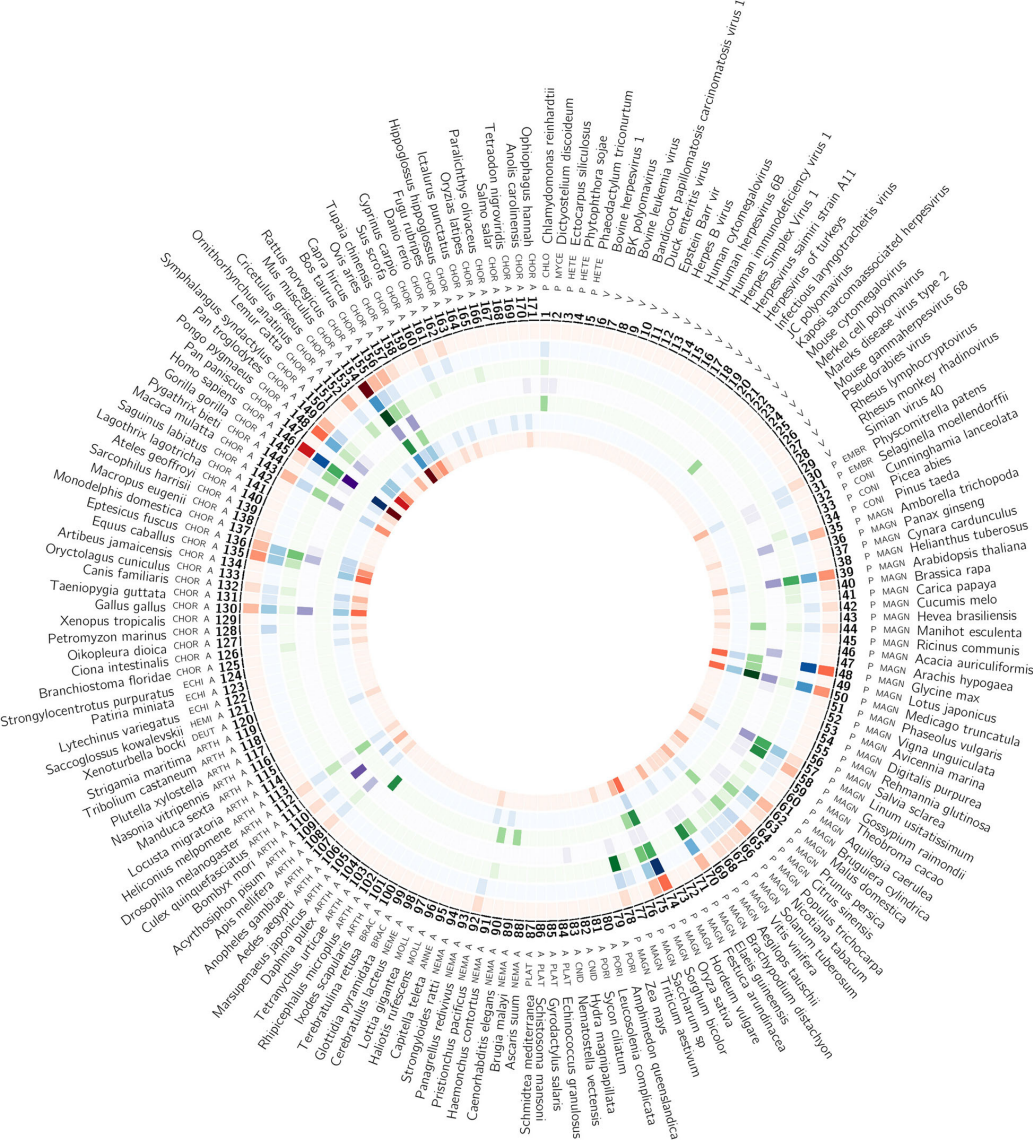
Comprehensive Circos plot depicting the frequency and distribution pattern of tandem repeats occurring in the miRNA precursors across different organisms. Outermost circle (I): The names of individual organisms selected for the study whose details are given in Additional file 1. Subsequent Inner circle (II):Phyla based categorization of organisms: Chlorophyta (Chloro), Mycetozoa (Myce), Heterokontophyta (Hete), Embryophyta (Embr), Coniferophyta (Coni), Magnoliophyta (Magn), Porifera (Pori), Cnidaria (Cnid), Platyhelminthes (Plat), Nematoda (Nema), Annelida (Anne), Mollusca(Moll), Nemertea (Neme), Brachiopoda(Brac), Arthropoda(Arth), Deuterostoma(Deut), Hemichordata (Hemi), Echinodermata (Echi) and Chordata(Chor). Subsequent Inner circle (III): Kingdom based classification of organisms: Protista (P), Plantae (P), Animalia (A) and Viruses (V). Subsequent Inner circle (IV): The corresponding serial numbers of organisms, as listed in additional file 1. Subsequent Inner circle (V): The relative count of dinucleotide type of SSRs in miRNA precursors. Subsequent Inner circle (VI): The relative count of trinucleotide type of SSRs in miRNA precursors. Subsequent Inner circle (VII): The relative count of tetranucleotide type of SSRs in miRNA precursors. Subsequent Inner circle (VIII): The relative count of pentanucleotide type of SSRs in miRNA precursors. Subsequent Inner circle (IX): The relative count of hexanucleotide type of SSRs in miRNA precursors. Subsequent Inner circle (X): The total count of miRNA precursors. Subsequent Inner circle (XI): The total count of SSR containing miRNA precursors

**Fig. 2:**
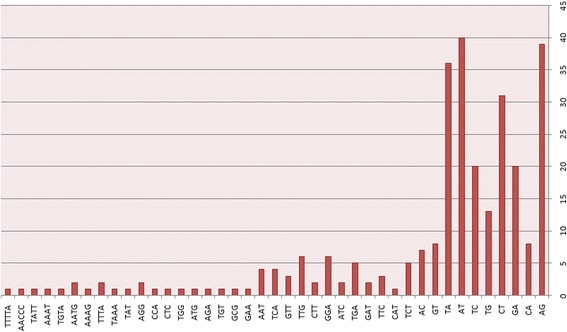
Distribution pattern of different types of SSR motif in the pre-miRNAs of *Arabidopsis thaliana.* The X-axis shows the different types of SSR motifs identified in the pre-miRNAs of *A.thaliana* and the Y- axis shows the relative count of each of the SSR motif identified

**Fig. 3:**
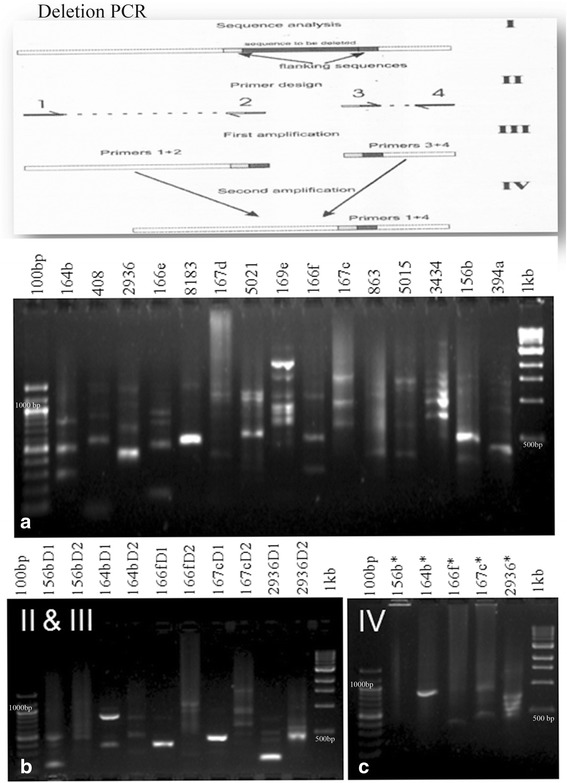
PCR products showing difference in size of the amplicons observed after Normal PCR with miRNA specific forward and reverse primers and Deletion PCR with forward and reverse primers designed to avoid SSR regions in pre-miRNAs. **a** Normal PCR with 15 sets of miRNAs (miR164b, miR408, miR2936, miR166e, miR8183, miR167d, miR5021, miR169e, miR166f, miR167c, miR863, miR5015, miR3434, miR156b and miR394a). **b** and **c** Primary and secondary deletion PCR with corresponding primer pair combinations; Lane A1, B1, C1:100 bp ladder, lane A17, B12, C7: 1 kb ladder, lane A2 to A16: Normal PCR products with miRNA specific forward and reverse primers; lane B2 to B11: Primary amplicons observed; lane C2 to C6: Final deletion PCR amplicons observed (* corresponds to deletion)

The SSRIT analysis of all the available miRNA precursors extracted from miRBase showed significant presence of SSRs which accounted to about 29.8%. The frequency and distribution pattern of SSRs varied extensively across different taxa analysed (see Additional file 1). SSR arrays were characterized as di, tri, tetra, penta or hexanucleotide based on the type of motif repeated in a sequence. Here, about 84.71% of SSRs were dinucleotide type of repeats, 12.5% were trinucleotide, 2.003% tetra, 0.544% penta and 0.181% were hexanucleotide type. When the relative count of SSRs bearing pre-miRNAs (the number of SSR bearing pre-miRNAs out of the total number of pre-miRNAs) were taken into consideration, *Homo sapiens* displayed the highest count, followed by *Mus musculus*, the least being *Cunninghamia lanceolata, Macropus eugenii, Lemur catta, Marsupenaeus japonicas, Strigamia maritima, Glottidia pyramidata, Leucosolenia complicata, Sycon ciliatum, BK polyomavirus, Bandicoot papillomatosis carcinomatosis virus, Herpesvirus saimiri strain A11, JC polyomavirus, Merkel cell polyomavirus* and *Simian virus 40*. The lesser count of SSR bearing pre-miRNAs may be due to lack of extensive miRNA characterisation studies in these organisms and hence these organisms cannot be completely demarcated. Similarly the relative count of SSR bearing pre-miRNAs were really scarce in a few organisms like *Avicennia marina, Phytophthora sojae* and *Terebratulina retusa*, still all of their pre-miRNAs revealed SSRs in their sequences showing 100% relative abundance of SSRs in miRNA precursors (Fig. 1). For a deeper and better understanding about the SSR motifs in the pre-miRNAs, we further focused our study in *Arabidopsis thaliana*, the model system. A closer examination of all the types of SSRs in the 325 reported pre-miRNAs of *Arabidopsis thaliana* exposed the significant presence of different types of SSR motifs. About 45% of pre-miRNAs in *A. thaliana* carried SSRs in their sequences of which 77% constituted dinucleotide type of SSRs, 19% trinucleotide, 3% tetranucleotide and 1% pentanucleotide type of SSRs. The distribution pattern of SSR types identified is shown in Fig. 2. Out of the 45% of SSR bearing pre-miRNAs, 7.5% of SSR bearing pre-miRNA showed transcription factors like SBP, MYB, NAC, HLZ, ARF, GRAS, ZF, BZIP, bHLH and WRKY as corresponding targets. A comparative analysis between normal PCR with miRNA specific primers and deletion PCR with primers designed to avoid SSR regions revealed a difference in the size of the PCR products as shown in Fig. 3. Five sets of miRNAs were further chosen based on certain criteria like the type of SSR motif, miRNAs with transcription factors as targets, the length of SSR repeat unit etc. The PCR profile showed either an absence or difference in size of the amplicons, which indicated the possible deletions of SSR regions in pre-miRNAs. Deeper focused studies give way to open up the potential significant roles for SSRs in pre-miRNAs.

### Clues for SSR involvement in shaping SREs for alternative splicing events

**Fig. 4:**
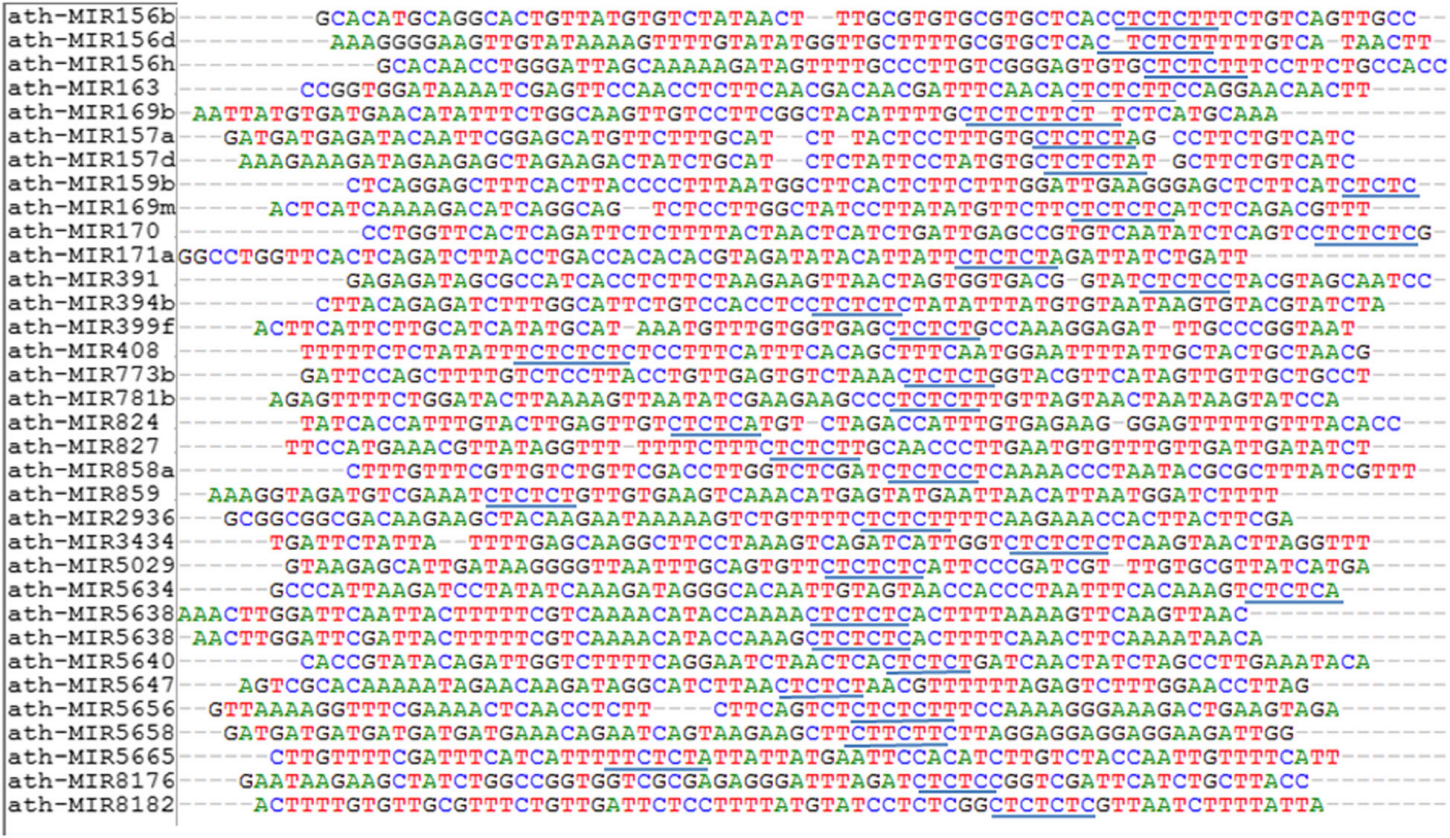
The CT dinucleotide SSR bearing pre-miRNAs in *Arabidopsis thaliana* with potential ISS activity. List of CT dinucleotide SSR bearing pre-miRNAs showing ISS activity. The blue underline indicates the position of ISS emphasizing the presence of CT motifs in such regions

The 149 SSR bearing pre-miRNAs identified in *A. thaliana* when subjected to RegRNA analysis, detected different functional RNA regulatory motifs. Among this, a most interesting and concurrent functional motif was Splicing Regulatory Element (SRE). The SREs were found to occur either in or near to the SSR motifs in pre-miRNA sequences. Out of the four SREs, the presence of Intron Splicing Silencers (ISS) in most of the CT/TC SSR motif type were noticeable. Such CT/TC motifs were well sustained in most of the members of conserved miRNA families like miR156 and 157. In miR854 family members, a trinucleotide SSR type GGA was found to be conserved with Exon Splicing Enhancer (ESE) like activity. A striking existence of two different types of SSR motifs adjacent to SREs like Intron Splicing Enhancers (ISE) [AG-ISE-CA] were also noticed among members of miR156 family. In addition to SREs, other functional RNA regulatory motifs associated with SSRs that were identified included Transcriptional Regulatory Motifs (TRM), Untranslated region motifs (UTRs), cis regulatory elements, noncoding RNA (ncRNA) hybridization regions, miRNA target sites etc. The AG motif was yet another SSR type which was conserved among miR8167 family members with potential function as TRMs. (see Additional file 2). This together with the observation that SSR motifs are consistently maintained even in the highly evolved Chordata and Magnoliophyta increased the chances of promising functions for such SSRs. The significant matches of SSRs in pre-miRNAs with SREs made us to check the possible role of SSRs in determining SREs required for the process of AS. For this, a computational based deletion analysis of SSR motifs in sequences of pre-miRNAs was carried out. Such tailored pre-miRNAs, when subjected to SRE prediction showed that, in the absence of certain SSRs the corresponding SREs were not detected (Table 1). Out of the four different SREs like ESE, ESS, ISE and ISS, the ISS and ESE elements were found to be the most affected when the SSR motifs were deleted. Among the SSR motifs, the CT/TC type was found to be the most prominent and consistent which were predicted as ISS sites (Fig. 4). If the CT/TC motif were deleted, the ISS sites were not detected in the corresponding pre-miRNA sequences. This initial result strengthened the possibility of SSR motifs to play a major role in shaping SREs to undergo AS.

**Table 1:** Computational deletion analysis of SSR motifs in pre-miRNAs of *A.thaliana* identifies potential role for SSRs in shaping SRE elements

Sl.no.	miRNA_Acc	SSR motif	ESE*	ESS*	ISE*	ISS*	ESE	ESS	ISE	ISS
1	ath-miR156a	ag			no ISE				ag-ISE	
2	ath-miR156b	ct				no-ISS				ct
3	ath-miR156d	ct				no-ISS				ct
4	ath-miR156e	ag			no-ISE				ag-ISE-ca	
		ct				no-ISS				ct
5	ath-miR156f	ag			no-ISE				ag-ISE-ca	
		ct				no-ISS				ct
6	ath-miR156g	ag			no ISE				ag-ISE-ca	
		ct				no-ISS				ct
		ct				no-ISS				ct
7	ath-miR156h	ct				no-ISS				ct
8	ath-miR156j	ag	no ESE				ESE-ag			
		ct				no-ISS				ct
9	ath-miR157a	ct				no-ISS				ct
10	ath-miR157b	ag	no ESE				ag-ESE			
		ct			ISE (gtgagac)	no -ISS				ct
11	ath-miR157c	at	no-ESE				ESE-at			
		ct				no-ISS				ct
12	ath-miR157d	ct				no ISS				ct
13	ath-miR159b	ct				no ISS				ct
14	ath-miR159c	ttc		no-ESS				ttc		
15	ath-miR163	ct				no-ISS				ct
16	ath-miR164b	ta	no-ESE				ta-ESE			
17	ath-miR165b	at	no-ESE				at-ESE			
18	ath-miR167c	ct			no-ISE				ISE-ct	
19	ath-miR167d	ca	no-ESE				ca-ESE			
		at			no-ISE				at-ISE	
20	ath-miR169b	ct				no-ISS				ct
		tct				no-ISS				tct
21	ath-miR169j	^a^g	no-ESE				ag			
22	ath-miR169l	^a^g	no-ESE				ag			
23	ath-miR169m	tc				no-ISS				tc
24	ath-miR169n	ag	no-ESE				ag			
25	ath-miR170	ct				no-ISS				ct
26	ath-miR171a	ca	no-ESE				ca			
		tc				no-ISS				tc
		ct				no-ISS				ct
27	ath-miR173	ag	no-ESE				ag			
28	ath-miR390b	ctt		no-ESS			ctt			
29	ath-miR391	ga	no-ESE				ga-ESE			
		tc				no-ISS				tc
		ta	no-ESE				ta-ESE			
30	ath-miR394a	at			no-ISE				AT-ISE	
		ta			no-ISE				at-ISE-ta	
31	ath-miR394b	ct				no-ISS				ct
32	ath-miR398b	ac	no-ESE				ac			
33	ath-miR398c	ac	no-ESE				ac			
34	ath-miR399b	at	no-ESE				ESE-at			
35	ath-miR399f	ct				no-ISS				ct
36	ath-miR408	ga	no-ESE				ga-ESE			
		tc				no-ISS				tc
37	ath-miR773b	ct				no-ISS				ct
38	ath-miR777	ta			no-ISE				ta	
39	ath-miR781b	ct				no-ISS				ct
40	ath-miR822	aat			no-ISE				aat	
41	ath-miR824	ct				no-ISS				ct
42	ath-miR827	ct			ISE (ttcttttg)	no-ISS		tatt		ct
43	ath-miR838	tct		ESS (tatttatta)				tct		
44	ath-miR847	tct		no ESS				tct		
45	ath-miR855	ta			ISE (ttctttta)				ISE-ta	
46	ath-miR857	^t^g		ESS (tagacat)				tg		
47	ath-miR858a	tc				no ISS				tc
48	ath-miR858b	at	no ESE				at			
49	ath-miR859	tc				no ISS				tc
50	ath-miR862	tg	no ESE				tg-ESE			
51	ath-miR863	tatt		no ESS				tatt		
52	ath-miR1886.1	ga			no ISE				ga	
53	ath-miR2111b	gaa	ESE (ggataca)				gaa			
		tga	ESE (ggataca)				tga-ESE			
54	ath-miR2936	ga	ESE (aagaagct)				ga-ESE			
		tc				no ISS				tc
		tc				no ISS				tc
		gcg	ESE (aagaagct)				gcg			
55	ath-miR2937	gt	no ESE				gt-ESE			
56	ath-miR3434	tc				no ISS				tc
57	ath-miR4240	at			no ISE				ISE-at	
58	ath-miR5025	at	no ESE				at			
59	ath-miR5027	tg			no ISE				tg	
60	ath-miR5029	ga	no ESE				ga-ESE			
		tc				no ISS				tc
		atg	no ESE				atg-ESE			
61	ath-miR5634	tc				no ISS				tc
62	ath-miR5638a	ct				no ISS				ct
		ac				no ISS				ac-ISS
63	ath-miR5638b	ga		no ESS				ga-ESS		
		ct				no ISS				ct
		ac				no ISS				ac-ISS
		ct				no ISS				ct
64	ath-miR5640	g^a^	no ESE				ga-ESE			
		ct				no ISS				ct
65	ath-miR5641	tct		no ESS				tct		
66	ath-miR5647	ct				no ISS				ct
		ct				no ISS				ct
		ct				no ISS				ct
67	ath-miR5648	ta	ESE (gaagaaa)				ESE-ta			
68	ath-MIR5651	at	no ESE				at-ESE			
		at	no ESE				ESE-at			
69	ath-miR5652	ga	no ESE				ga-ESE			
70	ath-miR5653	gttga		no ESS				ESS-gttga		
71	ath-miR5655	ag	ESE (gatgaca)				ag			
		ga	ESE (gatgaca)				ga			
		ag	ESE (gatgaca)				ag			
		tc	ESE (gatgaca)				tc			
		tgg			ISE				ISE-tgg	
		gga	ESE (gatgaca)				gga			
		cca	ESE (gatgaca)				cca			
72	ath-miR5656	tc				no ISS				tc
73	ath-miR5658	ctt			no ISE				ctt	
		agg				no ISS				ISS-agg
74	ath-miR5665	tat				no ISS				ISS-tat
75	ath-miR5997	agg	no ESE				agg			
76	ath-miR8166	ga	no ESE				ga			
77	ath-MIR8176	tc				no ISS				tc
78	ath-miR8177	ga	no ESE				ga			
79	ath-miR8182	ct				no ISS				ct

Note: The SSR bearing pre-miRNA and the SSR deleted pre-miRNA (*) shows difference in the detection of SREs like ESE, ESS, ISE and ISS

## Discussion

There exist different perspectives for SSRs like (1) hypervariable molecular marker which is well addressed and demonstrated by its utility as molecular marker during fingerprinting studies; (2) biological effects of SSRs in genes and (3) interplay between SSRs and miRNAs. The distribution of SSRs in genes is non-random presumably because they are supposed to have a variety of putative functions. Reports suggest that SSRs present in both coding and non-coding regions can affect gene expression [1]. SSRs in the 5’ UTR served as protein binding site there by regulating translation [16].SSR expansion in 3’UTR caused transcriptional slippage and produced expanded mRNA, which could accumulate as nuclear foci, and disrupt splicing, and other cellular function. Intronic SSR can affect gene transcription, mRNA splicing or export to cytoplasm. SSRs located in EST’s have a range of functions such as metabolic enzymes, structural and storage proteins, disease signaling, and transcription factors. A positive selection exists when the SSRs happen to occur near the transposons [17]. In Drosophila, a change in the 17-copy repeat of SSR in the *period* gene coding for Thr-Gly was found to affect the circadian rhythm maintenance [18]. Reporter assays predicted a putative enhancer-like function for TG repeats [19]. In rodents, the expression pattern of vasopressin 1a receptor (V1aR) is regulated by differences in SSR in the 5′ regulatory region which in turn affect the social behavior [20]. The expression of luciferase gene in reporter assays is directly proportional to the length of GA repeats thus can enhance the transcriptional output of a gene [21–23]. Repeats residing in the intronic regions also enhance gene expression like the TCAT repeat in the intron of Tyrosine Hydroxylase gene [24]. A significant enrichment of SSRs near the transcriptional start sites (TSS) (60 and 20% CCG and ACG found within 1 kb of TSS) was observed in humans. These examples indicate the importance and necessity of studying these SSRs and underline the fact that SSRs can be functional entities in the genome.

Out of the three perspectives, debates on the third perspective about the correlation between SSRs and miRNAs still exist. Very few reports suggest that SSRs are an important component of pre-miRNAs [10–12, 25, 26]. As the SSRs present in the genes are shown to have regulatory effects when associated with new miRNA candidates [27] and phenotypic effects [18, 20–24], we presume that the SSRs identified from pre-miRNAs in this study may have similar biological effects. Thus SSRs may be involved either in the biological function of miRNAs or its biogenesis. Here, the presence of SSR bearing or SSR related miRNAs across different taxa was well demonstrated and the incidence ratio in each of the respective organism was portrayed. A miniature platform detailing each and every aspect of SSRs like extent of occurrence, the type of motif etc. was successfully generated from the study. Previous reports suggest that Repeat-related miRNAs (RrmiRs) are those miRNA genes having at least 50% of repetitive elements or 100% in one of the associated mature miRNA sequences [28]. Recently identified RrmiRs in fungi and animals include the piwi associated small interfering RNAs (rasiRNAs) and heterochromatic small RNAs (hcRNAs), processed from long double stranded RNA precursors [29, 30].

A comparative study on the type of SSRs across the taxa showed that dinucleotide SSRs were the predominant type (84.71%), whereas hexanucleotide SSRs (0.181%) were the least. Moreover the CT and AG dinucleotide SSR motifs were found to be consistent among the members of highly conserved miRNA families like miR854, miR156 etc. This preference for dinucleotide type of SSRs in pre-miRNAs can be correlated to its probable functions. The frequencies of different repeats can vary considerably in different organisms. In humans, the A/T regions are more frequent and in *A.thaliana* GA/CT repeats are more [31]. The 5’UTRs of *Arabidopsis* are reported to have relatively more number of AG/CT repeats, whereas the 3’UTRs of humans and catfish possess more number of AC/GT repeats [32]. The frequency of (A/T) n was high in the intronic regions of different species, (AC/GT) n was high in primates, rodentia, mammalian, vertebrata, arthropoda, fungi etc. and CG/GC repeats were more in *C. elegans*, yeast and embryophyta. Among the dinucleotides identified from the current study, the CT motif was found to be the most frequent SSR. This can be correlated to its function. Earlier reports suggest that there is an increased probability for CT repeats to play a major role in the transcription of miRNA genes. CT repeats are reported to form non-B DNA that play important potential roles in gene transcription activation [33, 34]. Similar abundance of dinucleotide simple repeats like (CA) n and (TG) n were reported in the largest mir-467 family in mouse [27]. Each SSR generated might be the product of repeated mutations and cross-overs that might have occurred during the course of evolution. The resulting SSR type observed in a particular pre-miRNA may be the requisite of that particular pre-miRNA to undertake its specific function in the right way. We believe that ‘demand tunes the function by changing the sequence preference’. Also, the number (n) of times a particular type of SSR is repeated may or may not affect its putative function. One of the best reported examples is the fragile X syndrome (FXS), a triplet expansion disease (TRED), which is the most common neuropsychiatric and mental retardation disorder in humans [35]. When there is an expansion of a trinucleotide CGG repeat located in the 5’UTR of FMR 1 gene, to over 200 copies, it results in the deficiency of FMRP protein, which is required for normal neuronal development and plasticity.

The amplicon profile observed after deletion PCR is indeed a strong opening to study the effects of deletion of SSRs in pre-miRNAs. This together with the computational identification of SREs either in or near to SSRs made us to presume that SSRs are involved in shaping AS to generate variant miRNAs during stress environments. SREs are sequences in exons and introns that are important for constitutive splicing as well as alternative splicing. They function either as splicing enhancers or suppressors and affect splice site choice [15]. Our preliminary identification of SSRs either adjacent to or as SREs with splicing activity is the first implication for likely involvement of SSRs in Alternative Splicing (AS). A possible explanation for the presence of SSRs in pre-miRNAs is that the SSRs may fine tune the Alternative Splicing (AS) events in pre-miRNAs which contributes to different isoforms of miRNAs. As the miRNAs are tissue specific and developmental stage specific, each miRNA formed in response to a stress factor has a specific role. About 61 and 95% of intron containing protein-coding genes in *A. thaliana* and humans are reported to undergo AS [36, 37] and the stress responsive miRNAs were reported to be G/C rich in *A.thaliana* [38]. The miRNAs with UGUGU sequences are said to activate the targets associated with carcinogenesis in humans [39]. The correlation between AS and miRNAs was well demonstrated [40], where competition between the splicing machinery and the miRNA processing machinery comes into play. It is assumed that when the splicing machinery does not recognise the internal exon, the miRNA processing components bind to pre-miRNA splice junction, thereby leading to the formation of pre-miRNA and a skipped isoform. Whereas when the splicing components recognise the internal exon, pre-miRNA is not formed, instead an isoform bearing the internal alternative exon is formed. It is known that miRNAs are generated either from intergenic or intronic regions of coding or noncoding genes [41] and splicing and processing of intronic miRNAs may affect each other [34, 42]. A characteristic GU dinucleotide at the 5’end and AG at the 3’end is noticed for the canonical U2 type introns; whereas AU and AC dinucleotides at the 5′ and 3’ends were noticed for U12 type introns. This strengthens the intron retention process that may happen during AS events and this may also be another reason for occurrence of ‘tandem repeats’ in such hair-pin precursor sequences.

## Conclusions

The higher mutation rate of SSRs during recombination, polymerase slippage, DNA replication or repair, unequal crossing over etc. will finally end up with a change in number of repeat units of SSRs. This change may or may not become beneficial, depending on the incidence or position of these SSRs in a gene. The presence of SSRs in different locations that have an impact on genome strongly suggests that these SSRs should be considered significant and are not to be discarded as ‘nonsense’. Debates regarding the functional aspects of SSRs are never-ending. Those SSRs which are associated with miRNAs are speculated to have potential functions other than the conventional marker based assays. We speculate that there can be a tug of war between AS and miRNA biogenesis, which may in turn be affected, when there is a change in the number of repeat units (n) present in pre-miRNAs. All the three i.e. AS, SSRs and MIR genes are a complex interconnected network among which AS may be one of the crucial steps in miRNA biogenesis, which determine the formation of miRNAs in accordance with the external stress factors, whereas both AS and miRNA can be affected if a change in (n) occurs.

## Methods

All the available miRNA precursors (miRBase v.21) of different taxa {Chromalveolata, Metazoa, Mycetozoa, Viridiplantae and Viruses (see Additional file 1)} were extracted for the current study from public database, miRBase (www.mirbase.org). Among the miRNA precursors of the same genus but different species, the species with more number of miRNAs were included. Simple Sequence Repeat Identification Tool (SSRIT) [43] (http://archive.gramene.org/db/markers/ssrtool markers/ssrtool) was used to study the frequency, type and distribution pattern of SSRs in each individual sequences.

To identify the presence of different functional RNA motifs including Splicing Regulatory Elements (SREs), the SSR bearing pre-miRNA transcripts were subjected to an integrated web server RegRNA 2.0 analysis [44]. The pre-miRNAs bearing SSRs with SRE activity were selected for further analysis. To figure out whether the SSR motif had any effect in determining SRE elements, a computational deletion was carried out manually for each of the SSR sequence motif in their corresponding pre-miRNAs. Then the trimmed pre-miRNAs (after the deletion of SSR motifs) were again subjected to RegRNA analysis to understand the effects of SSRs. An experimental approach to generate deletion constructs for SSRs in pre-miRNAs was carried out using normal PCR with miRNA specific forward and reverse primers and deletion PCR with primers designed to avoid the SSR region in the pre-miRNAs. Out of 15 sets of miRNAs, five sets of miRNAs were chosen for the study which included miR156b, miR164b, miR166f, miR167c and miR2936. The details of the primer sequences are given in Additional file 3. Total RNA was isolated from in vitro seedlings of *Arabidopsis thaliana* wild variety (Col-0) using mirVana™ miRNA Isolation Kit (Ambion) according to manufacturer’s instructions. About 50 ng of total RNA was subjected to reverse transcription (RT) using the TaqMan MicroRNA Reverse Transcription Kit (Applied Biosystems™) in the presence of 0.15 μL of 100 mM dNTPs, 1.00 *μ*L of MultiScribe Reverse Transcriptase (50 U/μL), 1.5 *μ*L of 10X reverse transcription buffer, 0.19 *μ*L of RNase inhibitor (20 U/*μ*L) and 1.0 *μ*L of reverse primer in a total reaction volume of 15 *μ*L under cycling conditions: 16 °C for 30 min, 42 °C for 30 min, 85 °C for 5 min and a final 4 °C. The first strand cDNA was converted into dscDNA by carrying out a secondary PCR with 1.0 *μ*L of the template from first strand cDNA synthesis reaction, 1.0 *μ*L of 10X Advantage 2 PCR buffer (Clontech), 200 μM of each dNTPs (50X dNTP mix), 0.5 *μ*L of 10 *μ*M forward primer, 0.5 *μ*L of 10 *μ*M reverse primer and 0.5 *μ*L of 50X Advantage 2 Polymerase Mix in a total reaction volume of 10.0 *μ*L. The reaction was subjected to the following PCR conditions: 95 °C for 7 min, 35 cycles of 95 °C for 30s, 60 °C for 60s and 72 °C for 1 min, final extension at 72 °C for 10 min. Separate reactions were carried out for normal PCR and deletion PCR with all the constituents being the same except for primers used. The first strand and second strand cDNA PCR products were checked in 1.2% agarose gel to study the effects of deletion of SSRs in pre-miRNAs selected.

## Additional files

Additional file 1: The frequency and distribution pattern of SSRs in the pre-miRNAs across different taxa. (PDF 162 kb)

Additional file 2: RegRNA analysis of SSR bearing pre-miRNAs occuring in *A.thaliana* identified different functional RNA motifs. (PDF 149 kb)

Additional file 3: List of primer sequences used to carry out Normal and Deletion PCR for five sets of miRNAs selected. (PDF 96 kb)

## Abbreviations

ARF: Auxin response factor
bHLH: basic/helix-loop-helix
BZIP: Basic region/leucine zipper motif
GRAS: GRAS transcription factor
HLZ: Helix-leucine zipper motif
MYB: MYB transcription factor
NAC: No Apical Meristem domain
SBP: Squamosa promoter-binding protein domain
WRKY: WRKY transcription factor
ZF: Zinc finger
