# Supplementary material for: A deeper view into the significance of simple sequence repeats in pre-miRNAs provides clues for its possible roles in determining the function of microRNAs
Source: BMC Genet. 2018 May 9;19:29. doi: 10.1186/s12863-018-0615-x (PMC5941480; doi:10.1186/s12863-018-0615-x)
Supplement: Supplementary file 1 — The frequency and distribution pattern of SSRs in the pre-miRNAs across different taxa. (PDF 162 kb) [file 12863_2018_615_MOESM1_ESM.pdf]

Additional file 1: The frequency and distribution pattern of SSRs in the pre-miRNAs across different taxa										
**Kingdom**	**Phylum**	**Sl. No.**	**Complete list of organisms**	**Number of Dinucleotide type of repeats**	**Number of Trinucleotide type of repeats**	**Number of Tetranucleotide type of**	**Number of Pentanucleotide type of**	**Number of Hexanucleotide type of**	**Total number of miRNA precursors**	**Total number of SSR containing miRNA precursors**
Protista	Chlorophyta	1	*Chlamydomonas reinhardtii*	30	16	2	1	1	50	22
	Mycetozoa	2	*Dictyostelium discoideum*	2	3	0	1	0	17	5
	Heterokontophyta	3	*Ectocarpus siliculosus*	12	6	0	0	0	47	13
		4	*Phytophthora sojae*	2	0	0	0	0	2	2
		5	*Phaeodactylum triconurtum*	5	0	1	0	0	13	5
Viruses	Viruses	6	Bovine herpesvirus 1	4	2	0	0	0	10	5
		7	BK polyomavirus	0	0	0	0	0	1	0
		8	Bovine leukemia virus	1	1	0	0	0	5	2
		9	Bandicoot papillomatosis carcinomatosis virus 1	0	0	0	0	0	1	0
		10	Duck enteritis virus	7	0	0	0	0	24	5
		11	Epstein Barr vir	5	0	0	0	0	25	4
		12	Herpes B virus	10	1	0	0	0	12	7
		13	Human cytomegalovirus	5	0	0	0	0	15	4
		14	Human herpesvirus 6B	1	1	0	0	0	4	2
		15	Human immunodeficiency virus 1	3	0	0	0	0	3	2
		16	Herpes Simplex Virus 1	12	0	0	0	0	18	8
		17	Herpesvirus saimiri strain A11	0	0	0	0	0	3	0

		18	Herpesvirus of turkeys	10	1	0	0	0	17	8
		19	Infectious laryngotracheitis virus	0	1	0	0	0	7	1
		20	JC polyomavirus	0	0	0	0	0	1	0
		21	Kaposi sarcoma-associated herpesvirus	2	0	0	0	0	13	2
		22	Mouse cytomegalovirus	3	0	0	0	1	18	4
		23	Merkel cell polyomavirus	0	0	0	0	0	1	0
		24	Mareks disease virus type 2	11	0	0	0	0	18	9
		25	Mouse gammaherpesvirus 68	5	0	0	0	0	15	4
		26	Pseudorabies virus	5	1	1	0	0	13	7
		27	Rhesus lymphocryptovirus	2	2	0	0	0	36	4
		28	Rhesus monkey rhadinovirus	2	0	1	0	0	7	3
		29	Simian virus 40	0	0	0	0	0	1	0
Plantae	Embryophyta	30	*Physcomitrella patens*	136	17	0	1	0	229	104
		31	*Selaginella moellendorffii*	41	11	0	1	0	58	29
	Coniferophyta	32	*Cunninghamia lanceolata*	0	0	0	0	0	4	0
		33	*Picea abies*	13	4	1	0	0	40	13
		34	*Pinus taeda*	9	2	2	0	0	35	11
		35	*Amborella trichopoda*	156	21	1	2	0	124	83
		36	*Panax ginseng*	19	0	0	0	0	29	15
		37	*Cynara cardunculus*	20	2	0	0	0	48	11
		38	*Helianthus tuberosus*	3	1	1	0	0	16	3
		39	*Arabidopsis thaliana*	222	47	9	3	0	325	149
		40	*Brassica rapa*	87	8	3	0	0	96	45
		41	*Carica papaya*	33	4	3	0	0	79	27
		42	*Cucumis melo*	88	19	1	0	0	120	62
		43	*Hevea brasiliensis*	16	2	1	0	0	31	11
		44	*Manihot esculenta*	84	12	2	0	1	153	68
		45	*Ricinus communis*	39	5	1	0	0	63	29
		46	*Acacia auriculiformis*	6	0	0	0	0	7	4

	Magnoliophyta	47	*Arachis hypogaea*	13	0	0	2	0	23	11
		48	*Glycine max*	309	67	1	0	1	573	248
		49	*Lotus japonicus*	32	4	0	0	1	62	22
		50	*Medicago truncatula*	244	51	3	3	3	672	205
		51	*Phaseolus vulgaris*	9	1	0	1	0	8	4
		52	*Vigna unguiculata*	15	0	0	0	0	18	8
		53	*Avicennia marina*	6	0	0	0	0	2	2
		54	*Digitalis purpurea*	4	1	0	0	0	13	4
		55	*Rehmannia glutinosa*	8	0	0	0	0	32	7
		56	*Salvia sclarea*	7	2	0	0	0	18	7
		57	*Linum usitatissimum*	80	29	9	3	0	124	71
		58	*Gossypium raimondii*	160	16	10	1	0	296	121
		59	*Theobroma cacao*	24	10	1	1	0	82	35
		60	*Aquilegia caerulea*	25	8	2	0	0	45	17
		61	*Bruguiera cylindrica*	4	2	0	0	0	4	3
		62	*Malus domestica*	162	29	3	1	0	206	106
		63	*Prunus persica*	122	11	6	1	0	180	87
		64	*Citrus sinensis*	41	8	3	0	0	60	30
		65	*Populus trichocarpa*	154	24	6	0	1	352	132
		66	*Nicotiana tabacum*	132	11	2	1	0	162	69
		67	*Solanum tuberosum*	118	10	2	0	0	224	82
		68	*Vitis vinifera*	82	10	0	0	0	163	61
		69	*Aegilops tauschii*	66	5	6	1	0	88	44
		70	*Brachypodium distachyon*	189	39	10	1	0	317	139
		71	*Elaeis guineensis*	0	2	0	0	0	6	2
		72	*Festuca arundinacea*	9	6	0	0	0	15	8
		73	*Hordeum vulgare*	26	9	0	0	0	69	20
		74	*Oryza sativa*	317	86	8	1	2	592	243
		75	*Sorghum bicolor*	153	32	7	1	1	205	110
		76	*Saccharum sp.*	21	1	1	0	0	19	8
		77	*Triticum aestivum*	47	11	2	0	0	116	39
		78	*Zea mays*	133	25	11	1	0	172	90

	Porifera	79	*Amphimedon queenslandica*	6	1	0	0	0	8	6
		80	*Leucosolenia complicata*	0	0	0	0	0	1	0
		81	*Sycon ciliatum*	0	0	0	0	0	1	0
	Cnidaria	82	*Hydra magnipapillata*	2	0	0	0	0	17	1
		83	*Nematostella vectensis*	13	0	0	1	0	141	12
	Platyhelminthes	84	*Echinococcus granulosus*	4	0	0	0	0	23	4
		85	*Gyrodactylus salaris*	15	2	0	0	0	60	14
		86	*Schistosoma mansoni*	29	8	0	0	0	115	24
		87	*Schmidtea mediterranea*	25	5	0	0	0	148	27
	Nematoda	88	*Ascaris suum*	17	2	0	0	0	97	17
		89	*Brugia malayi*	29	4	1	0	1	115	27
		90	*Caenorhabditis elegans*	52	7	1	0	0	250	50
		91	*Haemonchus contortus*	28	2	2	0	1	188	30
		92	*Pristionchus pacificus*	97	10	0	0	0	354	72
		93	*Panagrellus redivivus*	20	3	0	0	0	200	20
		94	*Strongyloides ratti*	14	5	0	0	0	106	17
	Annelida	95	*Capitella teleta*	22	2	1	0	0	129	21
	Mollusca	96	*Haliotis rufescens*	1	0	0	0	0	5	1
		97	*Lottia gigantea*	13	1	0	0	0	59	12
	Nemertea	98	*Cerebratulus lacteus*	1	0	0	0	0	2	1
	Brachiopoda	99	*Glottidia pyramidata*	0	0	0	0	0	1	0
		100	*Terebratulina retusa*	1	0	0	0	0	1	1
		101	*Ixodes scapularis*	21	1	1	0	0	49	16
		102	*Rhipicephalus microplus*	6	1	0	0	0	24	5
		103	*Tetranychus urticae*	11	4	1	0	0	52	11
		104	*Daphnia pulex*	16	1	0	0	0	44	14
		105	*Marsupenaeus japonicus*	0	0	0	0	0	5	0
		106	*Aedes aegypti*	19	0	0	0	2	101	17

Animalia	Arthropoda	107	*Anopheles gambiae*	25	0	0	0	0	66	21
		108	*Apis mellifera*	97	19	2	2	0	254	94
		109	*Acyrthosiphon pisum*	16	2	0	0	0	123	15
		110	*Bombyx mori*	116	18	1	4	0	487	116
		111	*Culex quinquefasciatus*	23	1	0	0	0	74	18
		112	*Drosophila melanogaster*	90	15	4	1	1	256	74
		113	*Heliconius melpomene*	26	2	1	0	0	92	25
		114	*Locusta migratoria*	4	0	0	0	0	7	3
		115	*Manduca sexta*	31	4	0	0	0	98	29
		116	*Nasonia vitripennis*	13	1	0	0	0	53	12
		117	*Plutella xylostella*	29	9	1	0	0	133	25
		118	*Tribolium castaneum*	60	6	0	0	0	220	54
		119	*Strigamia maritima*	0	0	0	0	0	3	0
	Deuterostoma	120	*Xenoturbella bocki*	3	0	0	0	0	8	3
	Hemichordata	121	*Saccoglossus kowalevskii*	30	2	0	0	0	89	26
	Echinodermata	122	*Lytechinus variegatus*	10	2	0	0	0	50	12
		123	*Patiria miniata*	12	2	0	0	0	49	12
		124	*Strongylocentrotus purpuratus*	15	3	0	0	0	63	17
		125	*Branchiostoma floridae*	43	9	1	0	0	156	43
		126	*Ciona intestinalis*	70	9	2	0	0	348	68
		127	*Oikopleura dioica*	8	3	0	0	0	66	11
		128	*Petromyzon marinus*	56	22	0	0	0	244	68
		129	*Xenopus tropicalis*	41	4	0	0	0	192	38
		130	*Gallus gallus*	210	37	3	3	0	740	203
		131	*Taeniopygia guttata*	46	15	1	0	0	247	52
		132	*Canis familiaris*	133	14	2	0	0	502	122

	133	*Oryctolagus cuniculus*	5	0	0	0	0	12	5
	134	*Artibeus jamaicensis*	2	0	0	0	0	19	2
	135	*Equus caballus*	236	30	4	0	0	715	234
	136	*Eptesicus fuscus*	211	36	7	2	0	502	180
	137	*Monodelphis domestica*	84	3	0	1	0	460	73
	138	*Macropus eugenii*	0	0	0	0	0	3	0
	139	*Sarcophilus harrisii*	28	7	1	0	0	64	25
	140	*Ateles geoffroyi*	13	0	0	0	0	60	10
	141	*Lagothrix lagotricha*	7	0	0	0	0	48	7
	142	*Saguinus labiatus*	10	0	0	0	0	42	9
	143	*Macaca mulatta*	181	20	5	2	0	619	168
	144	*Pygathrix bieti*	5	0	0	0	0	11	5
	145	*Gorilla gorilla*	108	10	6	1	0	352	95
	146	*Homo sapiens*	495	74	8	6	0	1881	455
	147	*Pan paniscus*	27	2	0	0	0	88	20
Chordata	148	*Pongo pygmaeus*	330	40	4	2	0	642	316
	149	*Pan troglodytes*	169	21	3	1	0	655	158
	150	*Symphalangus syndactylus*	4	0	0	0	0	11	4
	151	*Lemur catta*	0	0	0	0	0	16	0
	152	*Ornithorhynchus anatinus*	145	14	4	0	0	396	121
	153	*Cricetulus griseus*	50	5	1	0	0	200	43
	154	*Mus musculus*	652	52	14	3	2	1193	418
	155	*Rattus norvegicus*	184	22	6	0	0	495	150
	156	*Bos taurus*	205	18	4	3	1	808	193
	157	*Capra hircus*	96	7	0	0	0	267	83
	158	*Ovis aries*	40	1	1	0	0	106	35
	159	*Tupaia chinensis*	28	2	0	0	0	177	27
	160	*Sus scrofa*	104	13	2	0	0	382	101

		161	*Cyprinus carpio*	38	5	0	0	0	134	37
		162	*Danio rerio*	87	10	1	0	0	346	80
		163	*Fugu rubripes*	28	5	0	0	0	131	28
		164	*Hippoglossus hippoglossus*	12	1	0	0	0	40	11
		165	*Ictalurus punctatus*	36	6	2	0	0	281	40
		166	*Oryzias latipes*	39	5	0	0	0	168	39
		167	*Paralichthys olivaceus*	5	0	0	0	0	20	5
		168	*Salmo salar*	48	7	0	0	0	371	50
		169	*Tetraodon nigroviridis*	26	3	0	0	0	132	25
		170	*Anolis carolinensis*	67	2	0	1	0	282	63
		171	*Ophiophagus hannah*	46	5	0	0	0	198	39
