# Supplementary material for: A deeper view into the significance of simple sequence repeats in pre-miRNAs provides clues for its possible roles in determining the function of microRNAs
Source: BMC Genet. 2018 May 9;19:29. doi: 10.1186/s12863-018-0615-x (PMC5941480; doi:10.1186/s12863-018-0615-x)
Supplement: Supplementary file 2 — RegRNA analysis of SSR bearing pre-miRNAs occuring in A.thaliana identified different functional RNA motifs. (PDF 149 kb) [file 12863_2018_615_MOESM2_ESM.pdf]

Additional file 2: RegRNA analysis of SSR bearing pre-miRNAs occuring in *A.thaliana* identified different functional RNA motifs.

Sl no.	Target genes	miRNA_Acc	ESE	ESS	ISE	ISS	UTR	TRM	CIS	ASS	NCRNA	MIRNA TARGET SITES
1	SBP domain	ath-miR156a	ESE-ACTGCT		AG-ISE							
2		ath-miR156b				CT						
3		ath-miR156c	ESE-ACTGCT		ISE-CA							
4		ath-miR156d			ISE-CA	CT						
5		ath-miR156e		CA-ESS	AG-ISE-CA	CT						
6		ath-miR156f			AG-ISE-CA	CT						
7		ath-miR156g			AG-ISE-CA	CT						
8		ath-miR156h	ESE-AG			CT						
9		ath-miR156i	ESE-ACAGCT			CT						
10		ath-miR156j	ESE-AG			CT						
11	SPL9, squamosa promoter binding protein-like 9	ath-miR157a				CT						
12		ath-miR157b	AG-ESE			CT						
13		ath-miR157c	ESE-AT			CT		AG				
14		ath-miR157d				CT		AAAG				
15	AGAMOUS-like 8	ath-miR159b				CT						
16	myb-like HTH transcriptional regulator family protein	ath-miR159c		TTC								
17	S-adenosyl-L-methionine-dependent methyltransferases superfamily protein	ath-miR163	ESE-ACTGCG			CT						
18	NAC (No Apical Meristem) domain	ath-miR164b	TA-ESE			CT						
19	PHV, ATHB9 | Homeobox-leucine zipper family protein	ath-miR165b	AT-ESE									
20	ARF6 | auxin response factor 6	ath-miR167c			ISE-CT	CT	AT		AG			

21	AT hook motif DNA-binding family protein	ath-miR167d	CA-ESE		AT-ISE		TA			GT		
22	NFYA5, NF-YA5 | nuclear factor Y, subunit A5	ath-miR169b				CT, TCT						
23	NFYA5, NF-YA5 | nuclear factor Y, subunit A5	ath-miR169c										
24	UNE8, ATHAP2B, HAP2B, NF-YA2 | nuclear factor Y, subunit A2	ath-miR169e									AT	
25	NF-YA8 | nuclear factor Y, subunit A8	ath-miR169j	AG									
26	HAP2C, ATHAP2C, NF-YA3 | nuclear factor Y, subunit A3	ath-miR169l	AG									
27	HAP2C, ATHAP2C, NF-YA3 | nuclear factor Y, subunit A3	ath-miR169m				TC						
28	AP2C, ATHAP2C, NF-YA3 | nuclear factor Y, subunit A3	ath-miR169n	AG									
29	|transposable element gene	ath-miR170				CT						
30	transposable element gene	ath-miR171a	CA			TC, CT						
31	TAS1C | TAS1C; other RNA	ath-miR173	AG									

32	Protein kinase superfamily protein	ath-miR390b	CTT										
33	ACA10, CIF1, ATACA10	ath-miR391	GA-ESE, TA-ESE			TC							
34	Galactose oxidase/kelch repeat superfamily protein	ath-miR394a			AT-ISE								
35	Galactose oxidase/kelch repeat superfamily protein	ath-miR394b				CT							
36	MATE efflux family protein	ath-miR398b	AC										
37	MATE efflux family protein	ath-miR398c	AC										
38	UBC24, ATUBC24, PHO2 | phosphate 2	ath-miR399b	ESE-AT										
39	UBC24, ATUBC24, PHO2 | phosphate 2	ath-miR399f				CT							
40	oxidoreductase, 2OG-Fe(II) oxygenase family protein	ath-miR408	GA-ESE			TC							
41	|transposable element gene	ath-miR419						TTGC					
42	FAD-dependent oxidoreductase family protein	ath-miR447b			ISE-GT								
43	GRV2, KAM2 | DNAJ heat shock N-terminal domain-containing protein	ath-miR773b				CT							
44	COP1-interacting protein-related	ath-miR777			ISE-TA								

45	SUVH6 | SU(VAR)3-9 homolog 6	ath-miR778						AT-TRM				
46	SWIB/MDM2 domain;Plus-3;GYF	ath-miR781b				CT						
47	Cysteine/Histidine-rich C1 domain family protein	ath-miR822	ESE-ACTGCT		AT-ISE; AAT-ISE							
48	AGL16 | AGAMOUS-like 16	ath-miR824				TC						
49	NLA | SPX (SYG1/Pho81/XPR1) domain-containing protein	ath-miR827				TC						
50	ATFRO3, FRO3 | ferric reduction oxidase 3	ath-miR837						TAAA				
51	O-fucosyltransferase family protein	ath-miR838		TCT								
52	transposable element gene	ath-miR847		TCT								
53	CYP81F4 | cytochrome P450, family 81, subfamily F	ath-miR849										
54	Single-stranded nucleic acid binding R3H protein	ath-miR854a	GGA									
55		ath-miR854b	GGA									
56		ath-miR854c	GGA									
57		ath-miR854d	GGA									
58		ath-miR854e	GGA									
59	transposable element gene	ath-miR855			ISE-TA							

60	Aminotransferase-like, plant mobile domain family protein	ath-miR857		TG								
61	TT2, ATMYB123, MYB123, ATTT2 | Duplicated homeodomain-like superfamily protein	ath-miR858a				TC						
62	TT2, ATMYB123, MYB123, ATTT2 | Duplicated homeodomain-like superfamily protein	ath-miR858b	AT									
63	F-box and associated interaction domains-containing protein	ath-miR859				TC						
64	ATDBR1, DBR1 | debranching enzyme 1	ath-miR862	TG-ESE									
65	Ulp1 protease family protein	ath-miR863		TATT						GT-TA		
66	Transducin/WD40 repeat-like superfamily protein	ath-miR1886.1			GA							
67	unknown protein	ath-miR1888b										
68	Galactose oxidase/kelch repeat superfamily protein	ath-miR2111b	GAA; TGA-ESE									
69	SRp34a, SR34a, At-SR34a | SER/ARG-rich protein 34A	ath-miR2936	GA-ESE; GCG			TC						

70	Protein of unknown function	ath-miR2937	GT-ESE									
71	Pseudouridine synthase family protein	ath-miR3434				TC		GTT-TRM				
72	transposable element gene	ath-miR4240			ISE-AT							
73	basic helix-loop-helix (bHLH) DNA-binding superfamily protein	ath-miR5014a							AA AT			
74	transposable element gene	ath-miR5025	AT									
75	Dihydropterin pyrophosphokinase / Dihydropteroate synthase	ath-miR5027			TG							
76	Unknown	ath-miR5029	GA-ESE; ATG-			TC						
77	AAO1, AO1, ATAO, AT-AO1, AOalpha, AtAO1 | aldehyde oxidase 1	ath-miR5631					AT					
78	unknown protein	ath-miR5634				TC						
79	Protein of unknown function	ath-miR5638a				CT; AC-ISS						
80		ath-miR5638b		GA-ESS		CT; AC-ISS						
81	Protein of unknown function	ath-miR5640	GA-ESE			CT						
82	unknown protein	ath-miR5641		TCT								
83	TTG2, ATWRKY44, WRKY44 | WRKY family transcription factor family protein	ath-miR5647				CT						
84	other RNA	ath-miR5648	ESE-TA					AT				

85	AT3G26440.1	ath-MIR5651	AT-ESE-AT									
86	Tetratricopeptide repeat (TPR)-like superfamily protein	ath-miR5652	GA-ESE									
87	Dihydroxyacetone kinase	ath-miR5653		ESS-GTTGA								
88	CIPK15 | CBL-interacting protein kinase 15	ath-miR5655	AG, GGA, CCA, GA, TC		TG-ISE; ISE-TGG							
89	MEF9 | mitochondrial editing factor 9 |	ath-miR5656				TC						
90	unknown protein	ath-miR5658			CTT	ISS-AGG						
91	TOR | target of rapamycin	ath-miR5664					TA					
92	RNA binding (RRM/RBD/RNP motifs) family protein	ath-miR5665				ISS-TAT						
93	transposable element gene	ath-miR5997	AGG									
94	ATNEK4, NEK4 | NIMA-related kinase 4	ath-miR5999										
95	Uncharacterized conserved protein	ath-miR8166	GA									
96	Major facilitator superfamily protein	ath-miR8167a						AG-TRM				
97		ath-miR8167b						AG				
98		ath-miR8167c						AG				
99		ath-miR8167d						AG				
100		ath-miR8167e						AG				
101		ath-miR8167f						AG				
102	AT4G22320.1	ath-MIR8176						AG				
103	IAA7, AXR2 | indole-3-acetic acid 7	ath-miR8177	GA									
104		ath-MIR8180					TA					

105	Arabidopsis thaliana protein of unknown function	ath-miR8182				CT						
106	NAD(P)-binding Rossmann-fold superfamily protein	ath-miR8183										TTC
