# Supplementary material for: A deeper view into the significance of simple sequence repeats in pre-miRNAs provides clues for its possible roles in determining the function of microRNAs
Source: BMC Genet. 2018 May 9;19:29. doi: 10.1186/s12863-018-0615-x (PMC5941480; doi:10.1186/s12863-018-0615-x)
Supplement: Supplementary file 3 — List of primer sequences used to carry out Normal and Deletion PCR for five sets of miRNAs selected. (PDF 96 kb) [file 12863_2018_615_MOESM3_ESM.pdf]

Additional file 3: List of primer sequences used to carry out Normal and Deletion PCR for five sets of miRNAs selected.

Sl. no	Oligo Name	Sequence (5'-3')	Number of bases	Tm
1	miR156bF	GCTCCTATCTACCATCCTCTCT	22	59.4
2	miR156bR	GCGTAAGCAAGCACCCACTTCCACA	25	75.2
3	miR164bF	GACATAAACAACACTCGCACTTT	23	61.9
4	miR164bR	CTCCTGTCTAATACTCGCTAAC	22	56.4
5	miR166eF	GCTAGAATATTGACGACTCACGGAT	25	65.2
6	miR166eR	ATACATCAAAACTCGGTTTGC	21	60.4
7	miR167cF	CTGAGGATCCAAACACAGAAAGT	23	63.3
8	miR167cR	CTATAATTCAAGCCCTCTCTGTCCA	25	65.2
9	miR2936F	AGCAAAGCTAATTATGGGTC	20	57.4
10	miR2936R	CAATCACTGCTTGTTGTTCA	20	59.9
11	miR156bDelF	CTCTGCCTGCTTGACCAAATTTGGCTTAGA	30	75.2
12	miR156bDelR	TCTAAGCCAAATTTGGTCAAGCAGGCAGAG	30	75.2
13	miR164bDelF	ATTTTGTGATATAGATTGAGTGTGATGATA	30	62.5
14	miR164bDelR	TATCATCACACTCAATCTATATCACAAAAT	30	62.5
15	miR167cDelF	ATATGATCATAGCTTACTAGGTCATGCTGG	30	66.6
16	miR167cDelR	CCAGCATGACCTAGTAAGCTATGATCATAT	30	66.6
17	miR166fDelF	TCTCATGATTATAACGTCGGACCAGGCTTC	30	73.7
18	miR166fDelR	GAAGCCTGGTCCGACGTTATAATCATGAGA	30	73.7
19	miR2936DelF	ATTTTCGAAAGCCTTACACAGACGATACCA	30	71.5
20	miR2936DelR	TGGTATCGTCTGTGTAAGGCTTTCGAAAAT	30	71.5

Note: Primers from 1 to 10 were used for Normal PCR; 11 to 20 were used for Deletion PCR
